# Evaluation of Atlas-Based Mobile Application in Undergraduate Teaching in Oral Histopathology

**DOI:** 10.3390/healthcare11142087

**Published:** 2023-07-21

**Authors:** Shuangshan Deng, Zucen Li, Xinyu Ma, Yali Wei, Ping Lyu, Yi Fan

**Affiliations:** 1 Department of Stomatology, West China School of Stomatology, Sichuan University, Chengdu 610041, China; dengshuangshan@stu.scu.edu.cn (S.D.); 2018151651143@stu.scu.edu.cn (Z.L.); 2019151640146@stu.scu.edu.cn (X.M.); 2 State Key Laboratory of Oral Diseases, National Clinical Research Center for Oral Diseases, Department of Cariology and Endodontics, West China Hospital of Stomatology, Sichuan University, Chengdu 610041, China; weiyali@stu.scu.edu.cn (Y.W.); lyuping@stu.scu.edu.cn (P.L.)

**Keywords:** dental education, questionnaire survey, teaching materials, online learning, mobile device

## Abstract

The utilization of mobile devices in education is a growing trend in various subjects. We developed the Dental and Maxillofacial Development Teaching Atlas App, and applied it to the learning process of oral histopathology. The aim of the current study was to investigate the educational effects of atlas-based mobile-assisted teaching in the field of dental medicine, and to suggest relevant improvements. The Dental and Maxillofacial Development Teaching Atlas App encompasses a wide range of atlases. It harbors various features, such as terminology definitions, student communications, and teacher–student interactions. By conducting questionnaires (70 students) and a quiz (68 students), we obtained students’ feedback, to evaluate the effects and application prospects of the WeChat applet. The questionnaire results indicate that students experienced a high level of satisfaction and support. Additionally, students participated in the quiz, with the experimental group exhibiting significantly higher average scores than the control group. The fill-in-the-blank questions, image recognition questions, and the total score all demonstrated statistically significant differences, while the terminology definition questions did not. The Dental and Maxillofacial Development Teaching Atlas App facilitates students’ utilization of fragmented time for learning, and demonstrates positive effects in enhancing students’ learning interests and proactiveness. It also holds promising potential for applications in other disciplines in the field of dental education.

## 1. Introduction

Dentistry is characterized by a tight connection between theoretical knowledge and clinical practice. Therein, oral histopathology is a fundamental discipline, serving as an important intellectual bridge. As it is a morphological, histological, and experimental science, the shortcomings of traditional modes of instruction for oral histopathology have emerged gradually. The resources of histological sections and microscopes are insufficient. The complex structures of tissue slices are always difficult for students to understand, and the specimens have limitations when it comes to including all the key points. Improving the teaching effectiveness and aiding student comprehension have become challenging tasks.

Advanced digital technologies have been implemented in dental education, including virtual microscopy [1,2] and virtual simulation experiment teaching centers [3,4]. It has been demonstrated that digitalization has the potential to promote a revolution in dental education, to cultivate talented dentists [5]. With the development of the global network and the utilization of mobile devices, e-learning can enhance the efficiency of study, and balance resources, including teaching materials and teaching staff [6,7]. The mobile application frees teaching and learning processes from time and geographical restrictions. Meanwhile, the integration of attractive visual effects with abundant information gives the mobile application the potential to enhance learning efficiency. The functional development of mobile applications can lead to a range of diverse uses, including online tests, reviews, and dialogue between teachers and students.

The introduction of mobile device-based electronic learning modes has significant implications for morphological dental education. While the innovations of technology offer assistance to faculty members, the latter are often hesitant to apply novel educational patterns due to concerns over the outcomes. Therefore, it is necessary to develop the curriculum through research, by evaluating the learning effects and feedback among students [8,9].

In this study, we designed the Dental and Maxillofacial Development Teaching Atlas App. The app contains atlases, encompassing illustrations, experimental slices, diagrams, immunofluorescence-staining images, hand-drawn graphs, and radiographic images. Meanwhile, functions such as terminology definitions, student communications, and teacher–student interactions make it convenient and efficient. Questionnaires and a quiz were utilized to assess the outcome. The application of the teaching-assistant app could function to stimulate learning initiatives, lead to a higher-quality dental education, and combat the scarcity of educational resources.

## 2. Materials and Methods

### 2.1. Materials and Participants

This study was conducted according to the guidelines set by the Declaration of Helsinki, and was approved by the Institutional Review Board of West China Hospital of Stomatology, Sichuan University (WCHSIRB-D-2023-139). All participants were students at West China School of Stomatology, Sichuan University. The textbook used was oral histopathology (eighth edition, published by People’s Health Publishing House). The software utilized was the Dental and Maxillofacial Development Teaching Atlas App.

For the questionnaire element of the study, in order to collect feedback on the use of the atlas applet from students at different levels, we selected students in their third, fourth, and fifth year of dental education, who had already studied oral histopathology. We first conducted a sample-size calculation based on the formula: n = p * (1 − p)/[E^2^/Z^2^ + p * (1 − p)/N]. The confidence interval was set at 95%, with N being 86 (28 students in the third grade, 28 students in the fourth grade, and 30 students in the fifth grade; the population size was 86). E was set to 0.05, and P was set to 0.5. The calculated value of n was 70. Stratified sampling was employed, using a random number table across the three grade levels, resulting in a total sample of 70 individuals (23 students from the third grade, 23 students from the fourth grade, and 24 students from the fifth grade). The students were advised to use the atlas applet more than 3 h per week for a total of 10 weeks, with no limit on the number of times they used it.

For the quiz element of the study, to minimize experimental bias, we selected students in their first and second year, who had not studied oral histopathology before. All students from the first and second year participated in a randomized controlled study, and were stratified and randomly assigned to their grade levels. Ultimately, the participants were randomly divided into two groups (n = 34 in each group): one group taught themselves Chapter II and Chapter III of oral histopathology only according to the textbook (the control group). The other group learned the same chapters with the help of the textbook and the atlas applet (the experimental group). The total study duration and study frequency of the two groups were consistent.

### 2.2. The Dental and Maxillofacial Development Teaching Atlas App

**Figure 1:**
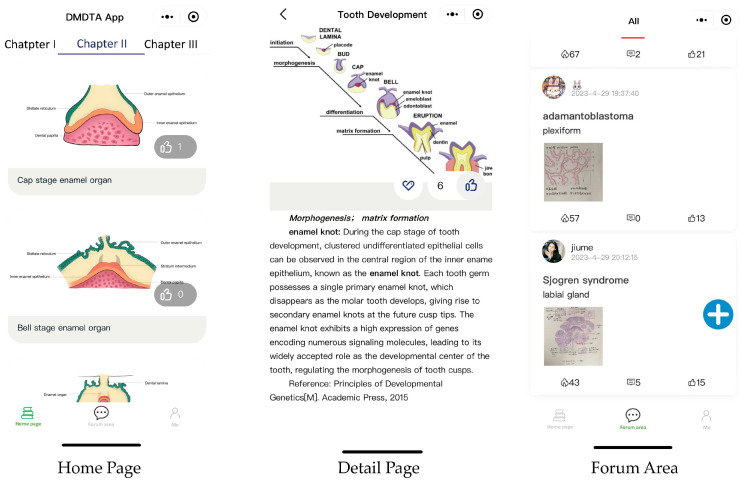
A sample of the page display of the atlas applet.

We have developed The Dental and Maxillofacial Development Teaching Atlas App (hereinafter referred to as the atlas applet) according to the content in the eighth edition of oral histopathology, published by People’s Medical Publishing House (Figure 1). In the development of the atlas applet, our team provided the overall framework and functional requirements for the applet. The applet development team adopted the Windows 10 operating system and JavaScript language, combined with the WeChat cloud development mode, to construct the atlas applet. Both parties maintained timely communication to optimize the service quality of the applet based on its performance. The entire process, from inception to deployment, took six months. The atlas applet is user-friendly, self-explanatory to students, and provides a rich collection of content that caters to students’ learning needs.

WeChat is an instant messaging app with an exceptionally high user adoption rate. Known for its diverse range of functionalities, WeChat applets serve as integrated applications within the WeChat platform, eliminating the need for additional downloads, and thereby facilitating effortless accessibility [10]. To utilize the atlas applet, students can register and log in using their student ID and password. The atlas applet comprises three main sections: the homepage, the forum area, and the personal section. The homepage serves as a comprehensive display of all the atlases, encompassing illustrations, experimental slices, diagrams, immunofluorescence-staining images, hand-drawn graphs, and radiographic images. All the images presented in the atlas applet are sourced diversely, including materials from textbooks, published literature, books, and student-generated illustrations. Currently, no clinical patient images are included. All images used in the atlas applet are obtained from open-access repositories, and proper citations have been provided to acknowledge their sources. Moreover, the atlas details page includes bilingual labeling in both Chinese and English, along with the corresponding definitions of relevant terminologies. This integration aims to meet students’ requirements for the precise alignment of knowledge with the atlas, facilitating a more effective grasp of the related concepts. The forum area is designed to foster communication among classmates, allowing students to showcase their hand-drawn graphs or raise questions. The personal section, titled ‘Me’, offers various features, such as a teacher Q and A, bookmarking, and browsing history. The teacher Q and A section enables students to ask questions on their personal computers, and receive timely responses from teachers.

### 2.3. Questionnaire

After 10 weeks of their using the atlas applet, we distributed an online survey questionnaire link to the participants. The questionnaire was designed by researchers, based on expert opinions and published literature [11,12]. These questions (Q1–15) were answered using a Likert scale of 1 representing “strongly disagree” and 5 representing “strongly agree” [13]. A Likert scale is used to gather information by providing participants with a set of answer options that span from one end of a spectrum to the other. It requires the participants to choose one option that best represents their opinion or experience [14].

As shown in Table 1, each question addressed different factors: Q1–5 explored ‘Satisfaction (F1)’, Q6–7 explored ‘Learning outcomes (F2)’, Q8–10 explored ‘Learning interests (F3)’, Q11–13 explored ‘Learning confidence and ability (F4)’, and Q14–15 explored ‘Appropriateness of the atlas applet as a learning evaluation tool (F5)’. All of these factors directly reflected the value and effectiveness of the atlas applet as a teaching aid.

### 2.4. Suggestions for the Improvement of the Atlas Applet

This part of the questionnaire consisted of five open-ended questions, as listed below. Although not mandatory, these questions collected students’ comments, and suggestions for improvements to the function of various parts of the atlas applet.

1. Do you have any suggestions on the terminology definitions and translations between Chinese and English?
2. Do you have any comments and suggestions on the comment collection and forum area?
3. Do you have any suggestions on the teacher inquiry settings?
4. If the hand-drawn images displayed in the atlas applet forum area could be mutually scored, would you like to upload your drawing to this interaction?
5. If the applet is applied to other dental disciplines, such as endodontics, oral and maxillofacial surgery, etc., which functions do you want to enrich?

### 2.5. Quiz

According to the teaching schedule of West China School of Stomatology, Sichuan University, this element lasted for one month. All participants took part in the offline closed-book quiz. The format of the quiz included fill-in-the-blank questions (20 points), image recognition questions (40 points), and terminology definition questions (40 points).

### 2.6. Data Analysis

SPSS 25.0 Statistics was used to perform data analysis. Cronbach’s alpha was used to assess the internal consistency of the questionnaire results. The non-parametric tests Mann–Whitney U test and Kruskal-Wallis test were performed, to analyze the qualitative data obtained from the questionnaire. Independent-samples *t*-tests were conducted and analyzed to determine the statistically significant differences between the experimental and control groups of scores in three sections, related to filling in the blanks, image recognition, and terminology definitions, in the quiz. The significance level was set as *p* < 0.05. 

## 3. Results

### 3.1. Questionnaire

**Figure 2:**
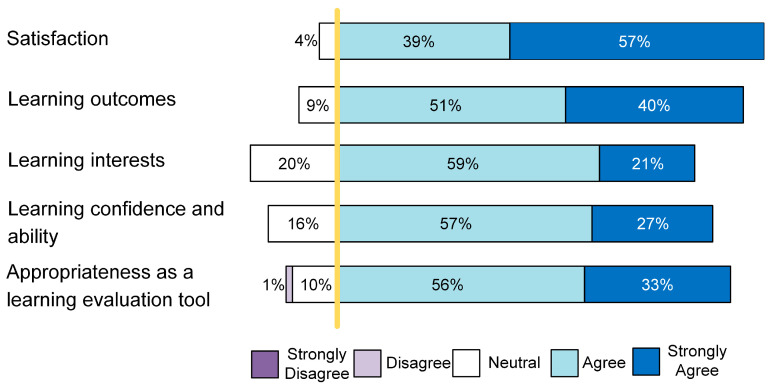
The specific selection of each factor.

In order to determine the reliability of the questionnaire, Cronbach’s alpha coefficient was calculated as 0.931. Cronbach’s alpha coefficient for each factor was as follows: 0.905 (F1), 0.887 (F2), 0.861 (F3), 0.866 (F4), and 0.892 (F5). The factors of the scales, ‘Satisfaction’, ‘Learning outcomes’, ‘Learning interests’, ‘Learning interests’, and ‘Appropriateness as a learning evaluation tool’ were examined. The results demonstrated that students generally reported positive opinions in respect of using the atlas applet to aid learning. The median, minimum, and maximum values of the questionnaire factors and total scores are presented in Table 2. All factors, ‘Satisfaction (F1)’, ‘Leaning outcomes (F2)’, ‘Learning interest (F3)’, ‘Learning confidence and ability (F4)’ and ‘Appropriateness as a learning evaluation tool (F5)’, demonstrated high average scores, and the mean total score of the scale was 65.00 [15]. In particular, ‘Satisfaction (F1)’ had a higher proportion of choices indicating ‘agree’ or ‘strongly agree’, and only ‘Appropriateness as a learning evaluation tool (F5)’ exhibited cases where the option of ‘disagree’ was selected (Figure 2).

**Figure 3:**
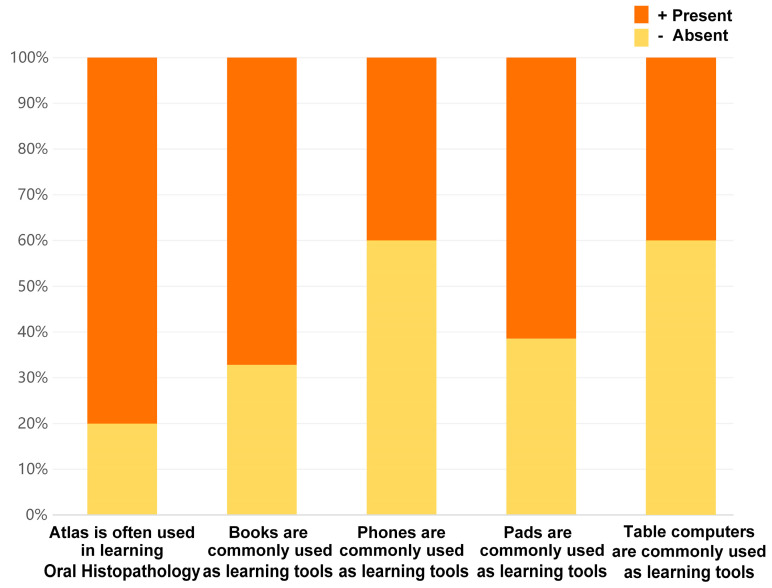
The use of various learning tools.

As shown in Table 3, there was no significant difference among students from the three different grades in the various factors. The majority of students opted to utilize the atlas as a preferred study tool for oral histopathology, while physical textbooks and pads were also popular choices among many students as their learning materials (Figure 3). Additionally, according to Table 4, it can be inferred that the preference for different devices did not affect students’ user experience of the atlas applet.

### 3.2. Opinions about the Improvement of the Atlas Applet

In the open-ended question section, the feedback revealed the following suggestions: 59 students (84.29%) recommended adding a keyword search function to locate specific atlases within the applet. The majority of students suggested incorporating a translation display/hide feature, and a terminology definition display/hide feature, to enhance the practicality of the applet. One student proposed the ability to add personal terminology definitions, enabling students to actively interact with, and adjust, the teaching content.

Regarding improvements to the forum section, 54 students (77.14%) believed that adding a categorization and bookmarking feature, along with a post search function, would be beneficial. Furthermore, there was considerable support for classifying forum posts based on their topic, with the goal of enriching and enhancing the flexibility of the forum section.

In terms of teacher–student interactions, 56 students (80%) expressed a desire for the functionality to send images and provide links to specific graphs within the applet. This would enable a more accurate description of their questions to teachers, and facilitate precise answers to student queries. 

When asked if they would be willing to participate in the hand-drawn graph interacting rating, 40 students (57.14%) expressed willingness to upload and evaluate graphs, while 18 students (25.71%) were willing to showcase but not evaluate, and 9 students (12.86%) showed interest in evaluating only. Three students expressed no interest in either activity. 

Regarding the inclusion of the atlas applet in other disciplines such as endodontics, 52 students (74.29%) expressed interest in the addition of case discussions. Moreover, 31 students (44.29%) expected the inclusion of quizzes, while 55 students (78.57%) expressed a desire for the display of clinical case images. Fifty students (78.57%) suggested the inclusion of step-by-step diagrams for clinical procedures, and real-time questionnaire feedback during classes was requested by 50 students (71.43%).

### 3.3. Quiz

Upon comparing the performance of the experimental group with that of the control group, it was found that the former showed improvements in all aspects, particularly in the fill-in-the-blank questions, image recognition questions, and total scores, with statistically significant differences. There was no statistically significant difference between the two groups in terms of the terminology definition questions (Table 5).

## 4. Discussion

As one of the fundamental disciplines in dental education, oral histopathology plays a crucial role as a foundation for future clinical practice. A command of the physiology and pathological structure of oral tissues is essential for the correct diagnosis and treatment of oral diseases. Atlases, as an intuitive summary of tissue anatomy and disease characteristics, could facilitate the learning initiatives of students. In this study, a dental and maxillofacial development atlas application has been developed. It takes advantage of mobile devices and various learning patterns, such as Chinese–English bilingual labeling and real-time interaction. It has served as a beneficial supplement to oral histopathology education, providing considerable assistance to students and teachers.

The results of the questionnaires verified that students spoke highly of the learning-aid function of the atlas applet. Considering the wide application of physical textbooks and pads, the preference for the atlas applet to serve as an additional tool was consistent and unchanging, reminding us that the atlas APP-assisted learning model has a low threshold of use, and is suitable for the majority of students. Meanwhile, the results suggested a predilection for the atlas, as well as for pads. There was a prominent tendency toward e-learning. The atlas applet is available for electronic equipment including smartphones, pads, and computers. Therefore, the Dental and Maxillofacial Development Teaching Atlas App emerged as being in demand, and having great potential.

Functions of the atlas applet involving bilingual labeling, corresponding definitions of terminologies, forum communication, and a teacher Q and A section were designed. The Q and A section allowed students to ask questions, and teachers could reply in time/on their personal computer. It was expected to enhance teacher–student interaction, resolve students’ doubts in a timely manner, supplement knowledge, and improve the learning effectiveness. The atlas applet, using mobile terminals as the carrier, conformed to the learning habits of contemporary students, and was more convenient than traditional paper-based atlases. The study provided constructive suggestions for the functional optimization of the atlas applet. The functions of the keyword search, personal terminology definition, categorization, and bookmarking aimed to offer a personalized and practical user experience. Regarding the demand for clinical competence training, updating the content of clinical case discussions and images, and step-by-step diagrams for clinical procedures were appealing. The hand-drawn graph interactive rating aroused interest, as well. This indicated that the display of hand-drawn graphs in the applet’s interactive section could enhance students’ initiative, and suggested a potential future direction for the atlas applet as a platform for showcasing drawing skills in competitions. 

Digital morphological course resources in dental education can visualize abstract concepts. Furthermore, they can encourage cooperation and communication between students and teachers, and mean that learning, evaluation, and feedback are not restricted by time or space. Students apply the app to make reasonable use of fragmented time. This learning pattern is in line with the trend of contemporary information development. It helps to build a learning society that can learn anytime and anywhere, and provides corresponding technical support in the development of contemporary stomatology talents. It can meet the principle of communal constructivism, which means that students learn with or from others, and contribute resources to the learning community [16]. 

However, there are some potential drawbacks to atlas-applet-aided dental education. Research has demonstrated that uncivil behaviors, such as posting meaningless, vague, and even rude responses, are prone to occur in online learning environments [17]. Meanwhile, students without technical capabilities can struggle to access the atlas applet. The atlas applet has the risk of crash or malfunction. Though this incident has not occurred during the test period, it depends on the network quality, number of people online, and so on. In addition, regarding the case discussions suggested by the subjects, the data protection of patients, and legal restrictions need to be taken into consideration. Regarding the limitations of our current study, we only evaluated the effect of the atlas applet for oral histopathology, while the atlas applet had great potential in the teaching of other courses. Moreover, the participants were confined to students in West China School of Stomatology, Sichuan University. Further investigations on the effect of applying the atlas applet in other regions and schools are encouraged. 

Further optimization will be incorporated into the project. The recommended functions are expected to be included, such as the keyword search, categorization, and bookmarking. The atlas applet can be incorporated into problem-based learning (PBL). The key notion of PBL is that learning begins with a problem, query, or question that the learner seeks to solve [18]. The atlas applet, with the convenience of independent study and self-testing, is a potential tool for PBL, resulting in a novel and effective model of instruction. Meanwhile, to advance the user experience, more regulations and decisions need to be reached, to create a conducive online learning environment, and launch the development of digital dental education.

In summary, our research established a Dental and Maxillofacial Development Teaching Atlas App, and assessed the user comments and effects, providing a novel potential dental educational technique.

## Figures and Tables

**Table 1:** Questions in the scale questionnaire.

Question	Score				
1. I am satisfied with the concept displayed in the atlas applet.	1	2	3	4	5
2. I like the atlas applet’s function of combining terminology definitions to assist in learning oral histopathology.	1	2	3	4	5
3. I like the atlas applet’s function of English–Chinese translation to assist in learning oral histopathology.	1	2	3	4	5
4. I like the atlas applet’s interactive comments function to assist in learning oral histopathology.	1	2	3	4	5
5. I like the atlas applet’s teacher–student interaction function to assist in learning oral histopathology.	1	2	3	4	5
6. I believe that using the atlas applet for learning has deepened my understanding and mastery of knowledge in oral histopathology.	1	2	3	4	5
7. I believe that using the atlas applet for learning has improved my learning efficiency.	1	2	3	4	5
8. I believe that I actively use the atlas applet in learning oral histopathology.	1	2	3	4	5
9. I am willing to continue using the atlas applet in my future studies.	1	2	3	4	5
10. I believe that using the atlas applet can increase learning interest in early scientific research training or graduate studies.	1	2	3	4	5
11. Based on the assisted teaching of the atlas applet and my understanding, I can have a clearer understanding of the relevant tissue structure.	1	2	3	4	5
12. I believe that the atlas applet’s functions, such as terminology definitions, English–Chinese translation, and so on can help me strengthen my mastery of professional terminologies in oral histopathology.	1	2	3	4	5
13. I believe that the hand-drawn images displayed in the atlas applet and the user interaction in the forum area can increase my confidence and the possibility of drawing images on my own.	1	2	3	4	5
14. I believe that using the atlas applet as a carrier for online assessment in the oral histopathology course is feasible and reasonable.	1	2	3	4	5
15. I believe that the atlas applet can continue to provide assistance in the learning of subjects such as endodontics and oral and maxillofacial surgery.	1	2	3	4	5

**Table 2:** Questionnaire factors and total scores.

Question	Factor	Median	Min–Max
Q1–Q5	Satisfaction	21.69	15.00–25.00
Q6–Q7	Learning outcomes	8.86	6.00–10.00
Q8–Q10	Learning interests	12.73	9.00–15.00
Q11–Q13	Learning confidence and ability	13.01	9.00–15.00
Q14–15	Appropriateness as a learning evaluation tool	8.71	5.00–10.00
Total score		65.00	45.00–75.00

**Table 3:** Questionnaire results of the three grades.

Grade	n (%)	F1 Median (Min–Max)	*p*	F2 Median (Min–Max)	*p*	F3 Median (Min–Max)	*p*	F4 Median (Min–Max)	*p*	F5 Median (Min–Max)	*p*
3	23 (32.86)	23.00 (18.00–25.00)	0.367	9.00 (8.00–10.00)	0.085	13.00 (11.00–15.00)	0.086	13.00 (11.00–15.00)	0.547	9.00 (6.00–10.00)	0.371
4	23 (32.86)	21.00 (15.00–25.00)		8.00 (6.00–10.00)		13.00 (9.00–15.00)		13.00 (9.00–15.00)		9.00 (6.00–10.00)	
5	24 (34.29)	22.00 (15.00–25.00)		8.50 (6.00–10.00)		12.50 (9.00–15.00)		13.00 (9.00–15.00)		9.00 (5.00–10.00)	

Note: F1, Satisfaction; F2, Learning outcomes; F3, Learning interests; F4, Learning confidence and ability; F5, Appropriateness as a learning evaluation tool.

**Table 4:** Questionnaire results regarding the use of learning tools.

Variable		n (%)	Total Score Median (Min–Max)	*p*
Atlas is often used in learning oral histopathology	+	56 (80.00)	65.39 (45.00–75.00)	0.632
	−	14 (20.00)	63.43 (45.00–75.00)	
Books are commonly used as learning tools	+	47 (67.14)	67.00 (45.00–75.00)	0.278
	−	23 (32.86)	65.00 (45.00–75.00)	
Phones are commonly used as learning tools	+	28 (40.00)	65.00 (46.00–75.00)	0.188
	−	42 (60.00)	65.00 (45.00–75.00)	
Pads are commonly used as learning tools	+	43 (61.43)	65.00 (45.00–75.00)	0.407
	−	27 (38.57)	68.00 (45.00–75.00)	
Table computers are commonly used as learning tools	+	28 (40.00)	67.00 (45.00–75.00)	0.588
	−	42 (60.00)	65.00 (46.00–75.00)	

Note: +: Present; −: Absent.

**Table 5:** Quiz score.

Test	Group	Score	F	t	*p*
Fill in the blanks	Control	15.18 ± 1.87	5.592	−3.512	0.001
	Experimental	17.02 ± 2.56			
Picture recognition	Control	25.18 ± 3.27	1.931	−2.839	0.006
	Experimental	28.06 ± 4.94			
Terminology definition	Control	32.41 ± 4.01	0.011	−1.083	0.283
	Experimental	33.50 ± 4.27			
Total score	Control	73.00 ± 5.87	3.795	−3.196	0.002
	Experimental	78.59 ± 8.33			

## Data Availability

All data generated or analyzed during this study are included in the manuscript.
